# 

**Published:** 1897-12

**Authors:** 


### First International Exhibition of Dentistry and Dental Surgery

An International Exhibition of Dentistry, Dental Surgery and Hygiene, under the auspices of a committee having amongst its members a great number of competent experts, will take place this year in September at the Marlborough Hall, Polytechnic Institute, Regent Street, London, W. Foreign exhibitors, who are not able to attend the exhibition, or who have no representative in London, can, if desired, be represented by the management and have their exhibits placed, arranged and attended to during the exhibition. All applications are to be addressed to the office of the exhibition, 18 Hart Street, W. L. A competent jury will award prizes of honor, diplomas of gold and silver medals, etc.

### NOTICES.

### National Association of Dental Technics.

The fifth annual meeting of the National School of Dental Technics will be held at the Palmer House, Chicago. December 29 and 30, of this year. Its programme will entitle it to even greater success than experienced at any time during its previous years of existence. Its leading paper, from Dr. G. V. Black, on "Instrument Nomenclature, with Reference to Instrumentation," will mark a historical period in methods of teaching the manual of cavity preparation.

D. M. CATTELL, *Sec'y-Treas.*
HENRY W. MORGAN, *Pres.*

### The Southern Dental Association.

This society, as a branch of the National Dental Association, will meet in St. Augustine, Florida, February 22d to the 25th, inclusive 1898. Active preparations are being made, and there is great encouragement that the meeting will be eminently successful. That will be a delightful time of the year to visit Florida, it is then in its beauty; and a visit to the quaint and historic city of St. Augustine is always a matter of great interest, it has many features and attractions possessed by no other city of this country, and this will be an opportune time to make it a visit. The headquarters of the meeting will be at the *Ponce De Leon Hotel*, a magnificent house, having scarcely an equal in the country.

